# Plasmacytoid Dendritic Cells Mediate the Regulation of Inflammatory Type T Cell Response for Optimal Immunity against Respiratory Chlamydia Pneumoniae Infection

**DOI:** 10.1371/journal.pone.0083463

**Published:** 2013-12-26

**Authors:** Antony George Joyee, Xi Yang

**Affiliations:** Laboratory for Infection and Immunity, Department of Medical Microbiology and Department of Immunology, Faculty of Medicine, University of Manitoba, Winnipeg, Manitoba, Canada; Boston University, United States of America

## Abstract

*Chlamydia pneumoniae (Cpn*) infection is a leading cause for a variety of respiratory diseases and has been implicated in the pathogenesis of chronic inflammatory diseases. The regulatory mechanisms in host defense against *Cpn* infection are less understood. In this study, we investigated the role of plasmacytoid dendritic cells (pDCs) in immune regulation in *Cpn* respiratory tract infection. We found that *in vivo* depletion of pDCs increased the severity of infection and lung pathology. Mice depleted of pDC had greater body weight loss, higher lung bacterial burden and excessive tissue inflammation compared to the control mice. Analysis of specific T cell cytokine production pattern in the lung following *Cpn* infection revealed that pDC depleted mice produced significantly higher amounts of inflammatory cytokines, especially TNF-α, but lower IL-10 compared to the controls. In particular, pDC depleted mice showed pathogenic T cell responses characterized by inflammatory type-1 (CD8 and CD4) and inflammatory Th2 cell responses. Moreover, pDC depletion dramatically reduced CD4 regulatory T cells (Tregs) in the lungs and draining lymph nodes. Furthermore, pDC-T cell co-culture experiments showed that pDCs isolated from *Cpn* infected mice were potent in inducing IL-10 producing CD4 Tregs. Together, these findings provide *in vivo* evidence for a critical role of pDCs in homeostatic regulation of immunity during *Cpn* infection. Our findings highlight the importance of a ‘balanced’ immune response for host protective immunity and preventing detrimental immunopathology during microbial infections.

## Introduction

*Chlamydia pneumoniae* (*Cpn*) is a an obligate intracellular bacterial pathogen that causes a broad range of respiratory diseases including bronchitis, obstructive pulmonary disease, sinusitis etc. Worldwide, the prevalence of chlamydial infections is very high. In particular, antibodies (Abs) specific for *Cpn* can be detected in the sera of up to 70% of healthy human beings, implying that most individuals in the general population have had exposure to these organisms [1]. Further, the spectrum of *Cpn* infection has been extended to its association with chronic inflammatory disorders such as asthma, cardiovascular and neurologic diseases [2]–[5]. The pathogenesis of these inflammatory conditions is considered to be immunopathologically mediated.

So far, there is no vaccine available for chlamydial infections. The development of an effective vaccine against *Cpn* has been a challenging task due to the incomplete understanding of the complex immunologic mechanisms during infection. Studies using mouse models of *Cpn* infection have shown that activation of a type-1 T cell response, especially CD8 T cells, and IFN-γ are required for host defense [6]–[8]. However, the precise immune mechanisms involved in host resistance or detrimental pathology during *Cpn* infection have not been fully elucidated. Specifically, the roles of different types of immune cells and their interactions and soluble components in immune responses during infection remain less understood.

Plasmacytoid dendritic cells (pDCs) are a unique leukocyte population implicated in a variety of immune responses including infections [9]. These cells are known for their ability to secrete type I interferon (IFN) in response to viruses. pDCs have been also reported to play key roles in allergy and asthma [10], [11], anti-tumor immunity [12] and responses to some non-viral pathogens [13]–[16]. While their protective role during several viral infections has been relatively well established, the functional role of pDCs and the mechanisms involved in immune response to bacterial infections remain largely unknown. In a *Listeria monocytogenes* infection model, depletion of pDCs resulted in decreased inflammation, enhanced organism clearance, and reduced mortality of mice [14]. A short study reported by Ang *et al* showed that pDCs play a role in controlling *Legionella pneumophila* infection and the protective effect was independent of IFNα production [15]. A recent study by Crother *et al* investigated the role of pDCs in *Cpn* infection and showed that depletion of pDCs during acute *Cpn* infection affected innate immune responses, with initially reduced inflammation and delayed bacterial clearance. However, during late stage of infection, the pDC depleted mice had impaired bacterial clearance and prolonged inflammation in the lungs [17]. On the other hand, FLT3L-induced increase in pDCs led to enhanced pulmonary inflammation during acute *Cpn* infection. The findings by Crother *et al* showed the effect of pDCs in contributing to the innate immune responses during *Cpn* infection [17], however, the immunological events associated with the subsequent development of inflammation and pathology during infection remained unclear. More importantly, role of pDCs in modulating adaptive T cell immunity and the underlying regulatory mechanisms contributing to host defense against *Cpn* infection still remain to be understood. Understanding the precise nature of cellular immune responses following *Cpn* infection leading to protection or pathology is necessary, in consideration of the association of *Cpn* infection with chronic inflammatory airway diseases such as COPD, asthma etc.

In the present study, we investigated the role of pDCs and the mechanism by which they contribute to host resistance following *Cpn* infection. We found that pDCs are activated in the lungs following *Cpn* infection. Further, mice depleted of pDCs succumbed to increased severity of infection with higher bacterial loads as well as exacerbated lung pathological responses. Moreover, pDC activation following *Cpn* infection enhanced CD4 Tregs/IL-10 production and mediated the regulation of T cell responses for optimal immunity against infection. Overall, our findings showed that pDCs play a critical role in homeostasis for host protection during respiratory *Cpn* infection.

## Materials and Methods

### Mice

C57BL/6 mice were purchased from Charles River Canada (Montreal, Canada) The animals were maintained at a pathogen-free animal care facility at the University of Manitoba. Eight to 10-week-old mice were used in the study. All experiments were done in compliance with the guidelines issued by the Canadian Council of Animal Care, and the animal protocol was approved by the institutional ethical committee (#06-042).

### Bacterial Strain, Mouse Infection and Quantitation of *in vivo* Bacterial Loads

The culture and purification of *Cpn* (AR-39 strain) and infectivity determination in HL cells were performed as described previously [18]. Highly purified *Cpn* elementary body (EB) preparations were obtained by renografin gradient separation. A sonicated killed preparation of EBs (SK-EB) was used for *in vitro* restimulation assays [18]. Mice were infected intranasally with 3×10^6^ inclusion-forming units (IFUs) of *Cpn* in 40 µl of PBS following mild sedation. Mice were euthanized at specified time points post infection (p.i.), and the lungs were aseptically collected and processed for quantitative assessment of bacterial loads in the lungs [18].

### Histological Analysis

For histological analysis, lung were collected following infection, fixed in 10% buffered formalin and embedded in paraffin as described previously [18]. The tissue sections were stained with hematoxylin and eosin, and the histologic changes and cellular infiltration were assessed by light microscopy.

### pDC Isolation, Cytokine Production Analysis and *in vivo* Depletion

Lung single-cell suspensions were prepared essentially as described [18]. pDCs were isolated using mPDCA microbeads and MACS columns (Miltenyi Biotec) as per manufacturer’s instructions. After consecutively passing through two columns, the collected pDC preparations showed >95% purity. pDCs isolated from different groups of mice were cultured in complete RPMI-1640 medium in the presence of SK-EB (10^4^ IFUs) in 96 well plates at 1×10^6^ cells/well for 72 h and the supernatants were measured for IL-12p40 and IL-12p70 by ELISA as previously described [19]. For analysing IFNα production, cells were stimulated in the presence of SK-EB and monensin (eBioscience) for 5 h in complete RPMI 1640 medium, washed and subjected for intracellular staining and flowcytometry analysis. For in* vivo* depletion of pDCs, mice were injected i.v. with 100 µg/mouse of functional grade anti-mPDCA-1 Ab clone JF05-1C2.4.1 (Miltenyi Biotech, Auburn CA) or control Rat IgG2b Ab a day before *Cpn* infection and at every two days interval after inoculation until the animals were sacrificed. Depletion of pDCs was about 90–95% as evaluated by FACS analysis (not shown). Mice were killed at designated days after infection and analysed for lung bacterial loads and pathology described above.

### Flow Cytometry Analysis

For analyzing expression of surface costimulatory molecules, freshly isolated lung pDCs were stained using anti-mPDCA-allophycocyanin, anti-CD11c-PE, anti-MHC class II-FITC (I-A/I-E), anti-CD40-FITC, anti-CD80-FITC, anti-CD86-FITC or with respective isotype controls (eBioscience). IFNα production by pDCs was analysed by intracellular staining using anti-mouse IFNα-FITC Ab (PBL Biomedical). For CD4+Foxp3+ Treg staining, lung or lymph node cells were first stained for surface markers CD3 (anti-CD3-FITC) and CD4 (anti-CD4-PerCP), fixed and permeabilized using Fix/Perm buffer (eBioscience, San Diego, CA) and stained intracellularly for Foxp3 (anti-Foxp3-PE) according to manufacturer’s (eBioscience) instructions for Foxp3 staining. Specific analysis for Treg IL-10 production was performed by intracellular cytokine staining using anti-IL-10-allophycocyanin (eBioscience). Sample data were collected using a FACSCalibur flow cytometer (BD Biosciences), and the data were analyzed using WinMDI software, version 2.8 (Scripps Research Institute, La Jolla, CA). To analyse T cells, macrophages, DCs, and granulocytes in the lungs, total lung cells were stained using Abs against markers CD3, F4/80, CD11c, CD11b, and Gr-1 (eBioscience) and analyzed by flow cytometry. Lung alveloar macrophages were identified as F4/80+CD11c+ cells, conventional DCs (cDC) as F4/80−CD11c+ cells and granulocytes as F4/80−CD11c− Gr-1+ cells.

### *In Vitro* Restimulation Assays and Cytokine Measurements

Single-cell suspensions were prepared from draining (mediastinum) lymph nodes collected from *Cpn* infected mice and cultured at a concentration of 5.0×10^6^ cells/ml, alone or with SK-EB (10^5^ IFUs/ml). After incubation for 72 hours in a 5% CO_2_ atmosphere, the culture supernatants were collected and analyzed for the cytokines, TNF-α, IFNγ, IL-4 and IL-10 by ELISA.

### T Cell Intracellular Cytokine Staining

The specific cytokine production by CD8 and CD4 T cells was analyzed by intracellular cytokine staining and flowcytometry as described [19], [20]. Briefly, single-cell suspensions were prepared from lung and the cells were stimulated with PMA (20 ng/ml) and ionomycin (500 ng/ml) and incubated for 5 h in complete RPMI 1640 medium at 37°C in the presence of Brefeldin A (eBioscience). Cell surface staining for T cell markers CD3, CD8 and CD4 was performed first, followed by fixation and permeabilization using IC fixation and permeabilization buffers (eBioscience) as per manufacturer’s instructions. The cells were further stained intracellularly for cytokines using allophycocyanin or PE labeled anti-TNF, anti-IFNγ, anti-IL-4 and anti-IL-10 (eBioscience) or with corresponding isotope control Abs and analyzed by flow cytometry.

### pDC- CD4 T Cell Co-culture

The ability of pDCs to activate *C. pneumoniae* Ag-specific T cells and to induce Treg response was assessed using a pDC-T cell co-culture system. CD4 T cells were isolated from the spleens of *Cpn*-immunized mice using negative selection by depleting non-T cells [19]. pDCs isolated from *Cpn* infected or uninfected mice were co-cultured with purified CD4 T cells (pDC/CD4 T cell ratio, 1∶10) in 96 well plates in the presence of SK-EB (10^4^ IFUs/ml) in complete RPMI medium for 72 h. Subsequently, CD4 T cells were analysed for IL-10 and Foxp3 by intracellular staining as detailed above.

### Statistical Analysis

Data were analyzed using unpaired, two-tailed Student’s *t* test (GraphPad Prism software, version 4, Graph Pad, San Diego, CA). A *p* value less than 0.05 was considered significant.

## Results

### pDCs are Activated Following *Cpn* Infection

**Figure 1:**
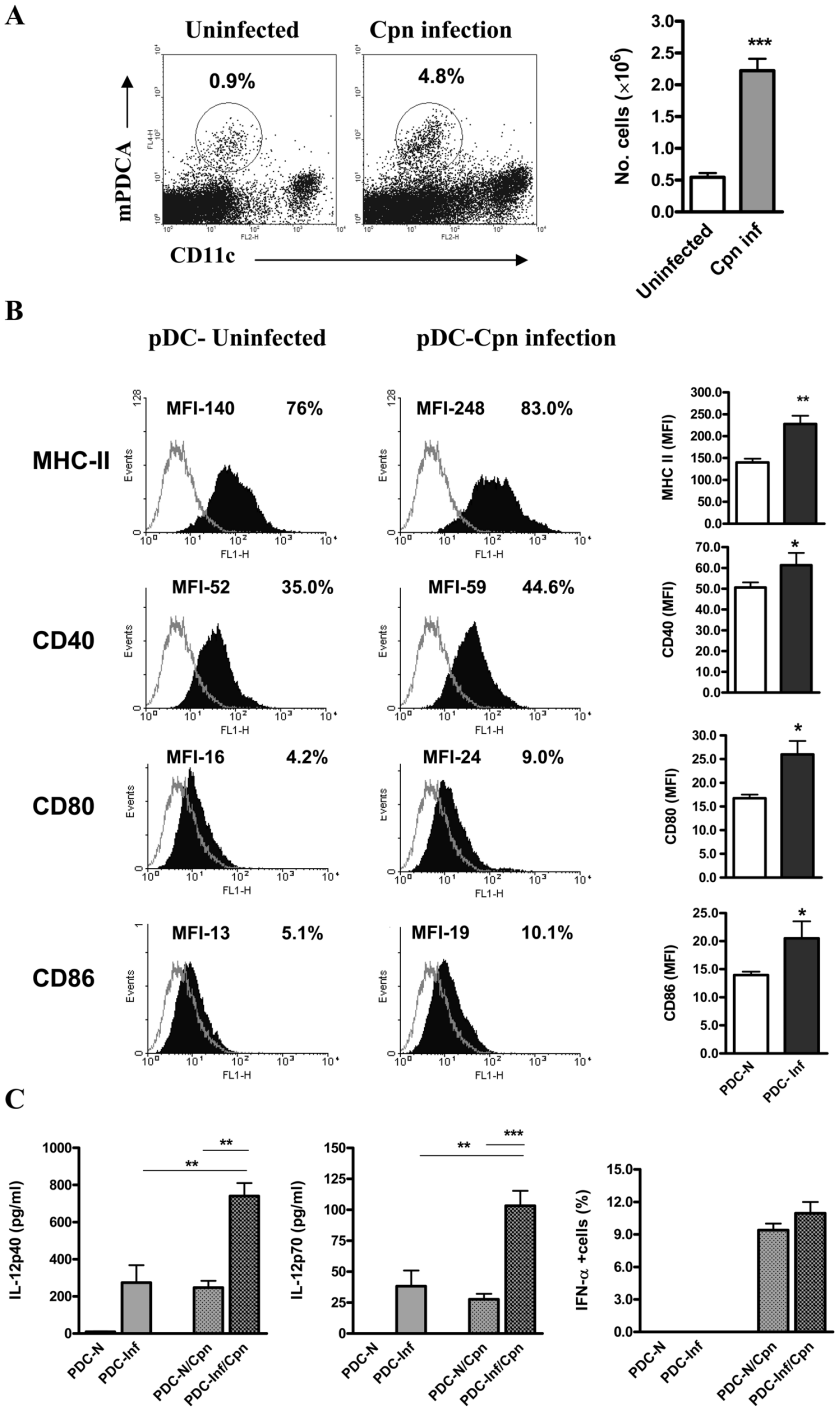
Activation of pDCs following *Cpn* infection. C57BL/6 mice were intranasally infected with *Cpn* (3×10^6^ IFUs). Mice were killed at day 9 p.i., and lungs were aseptically collected. To analyse pDCs, lung cells were stained and analysed by flowcytometery as decribed in the *Materials and Methods*. pDCs were identified as mPDCA+ CD11c ^int/lo^ cells. ***A,*** pDC expansion after *Cpn* infection. Shown are representative dot plots with the percentages of pDCs in *Cpn* infected mice in comparison with uninfected mice (left) and the graphical summary for the absolute numbers of pDCs in the lungs. Total pDC number per mouse lung was calculated as % mPDCA+ CD11c ^int/lo^ cells x total number of cells per mouse lung/100. ***B,*** Expression of MHC II and costimulatory molecules CD40, CD80 and CD86 on gated pDCs (filled histograms) and isotype control (dotted line) are shown. The mean fluorescence intensity (*left*) and the percentages of positive cells (*right*) are indicated. ***C,*** pDCs purified (as described in *Materials and Methods*) from *Cpn* infected (PDC-Inf) and uninfected mice (PDC-N) were cultured in the presence or absence of *Cpn* (SK-EB). IL-12p40, IL-12p70 in the 72 hrs supernatants were measured by ELISA. IFNα production by pDCs was analysed by intracellular staining and the graph shows the percentages of IFNα producing cells. Results are shown as mean ± SD. At least three independent experiments with four mice in each group were performed and one representative experiment is shown. *, p<0.05, and ***, p<0.001, Student’s t test.

We first tested whether pDCs showed an expansion following infection with *Cpn*. We found about 5 fold-increase in the frequency and numbers of pDCs in the lungs of *Cpn* infected mice (day 9 p.i.) (Figure 1A). Next, we examined the expression of surface costimulatory molecules on pDCs. The pDCs from *Cpn* infected mice showed higher expression of MHC II, CD40, CD80 and CD86 than the uninfected mice (Figure 1B). Further, we analysed the cytokine production by pDCs after infection. We found that pDCs from *Cpn* infected mice produced significantly higher IL-12 p40 and IL-12p70, whereas IFNα production was similar compared to that from uninfected mice (Figure 1C). Taken together, these results showed that pDCs are expanded and activated following *Cpn* infection with a specific pattern of cytokine production.

### pDC Depletion Leads to Increased Susceptibility to *Cpn* Infection with Severe Tissue Pathology

**Figure 2:**
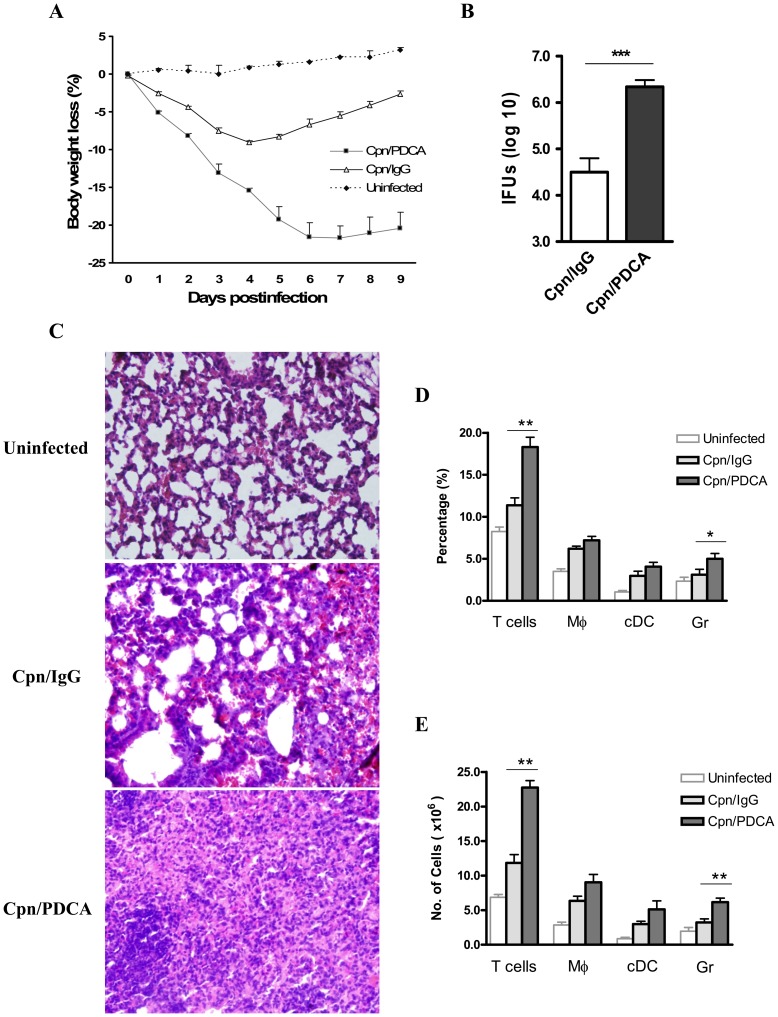
pDC depletion leads to increased bacterial loads and tissue inflammatory changes in the lungs. Mice were depleted of pDCs using anti-mPDCA Ab prior to and during the course of infection as described in the *Materials and Methods.* Control group mice received Rat IgG2b Ab. Following infection, the animals were monitored everyday for body weight changes (**A**). At day 9 p.i., the mice were sacrificed and the lungs were collected and quantitative assessment of bacterial loads was performed as described in *Materials and Methods*. ***C,*** Increased tissue pathological response in the lungs of pDC depleted mice compared to control mice as analysed by H&E staining. ***D &E,*** Shown are the graphs depicting differences in the percentage and absolute numbers of lung T cells (CD3+), Mφ, alveolar macrophages (F4/80+CD11c+ cells), cDCs, conventional DCs (F4/80−CD11c+ cells) and Gr, granulocytes (Gr-1+ F4/80−CD11c− cells). Results are shown as mean ± SD. Results of one representative (of three) experiment with four mice in each group are depicted. *, p<0.05, and ***, p<0.001.

To investigate the functional effect of pDCs in host immune response against *Cpn* infection, mice were depleted of pDCs and the infection outcome was evaluated. We found that, compared with the sham-treated mice, pDC depleted mice showed greater body weight loss and succumbed to severe disease. At day 9 p.i., these mice showed a loss of more than 20% of their bodyweights, whereas the control mice almost recovered their original body weight (Figure 2A). Next, we quantified the *in vivo Cpn* growth in the lungs. The pDC depleted mice showed significantly higher bacterial loads in their lung (nearly 100-fold higher) than the sham treated mice (Figure 2B). The histological analysis of lung tissues (day 9 p.i.) showed severely exacerbated tissue pathology in the pDC depleted mice, consistent with the higher bacterial burden in their lungs. There was a severe disruption of alveolar architecture, largely characterized by very diffused and massive cellular influx. In contrast, the sham-treated mice displayed much less lung pathological changes with reduced cellular infiltration in a more localized fashion, showing signs of recovery from infection (Figure 2C). To analyze the nature of cellular infiltration, lung cells from pDC depleted and control mice were examined for changes in the proportion and numbers of lymphocytes, macrophages, cDCs and granulocytes (neutrophils/eosinophils) following *Cpn* infection. We found that, notably the lungs of pDC depleted mice showed significant increase in T cells and granulocytes compared to that in the sham-treated mice (Figure 2D&E). Additionally, there was a nonsignificant trend of increase in alveolar macrophages and cDCs in the pDC depleted mice compared to the sham-treated mice (Figure 2D&E). Together, these data showed that pDC deficiency rendered mice more susceptible to *Cpn* lung infection with increased *in vivo* pathogen growth and extensive lung pathology.

### pDC Deficiency Leads to Altered Cytokine Production Following *Cpn* Infection

**Figure 3:**
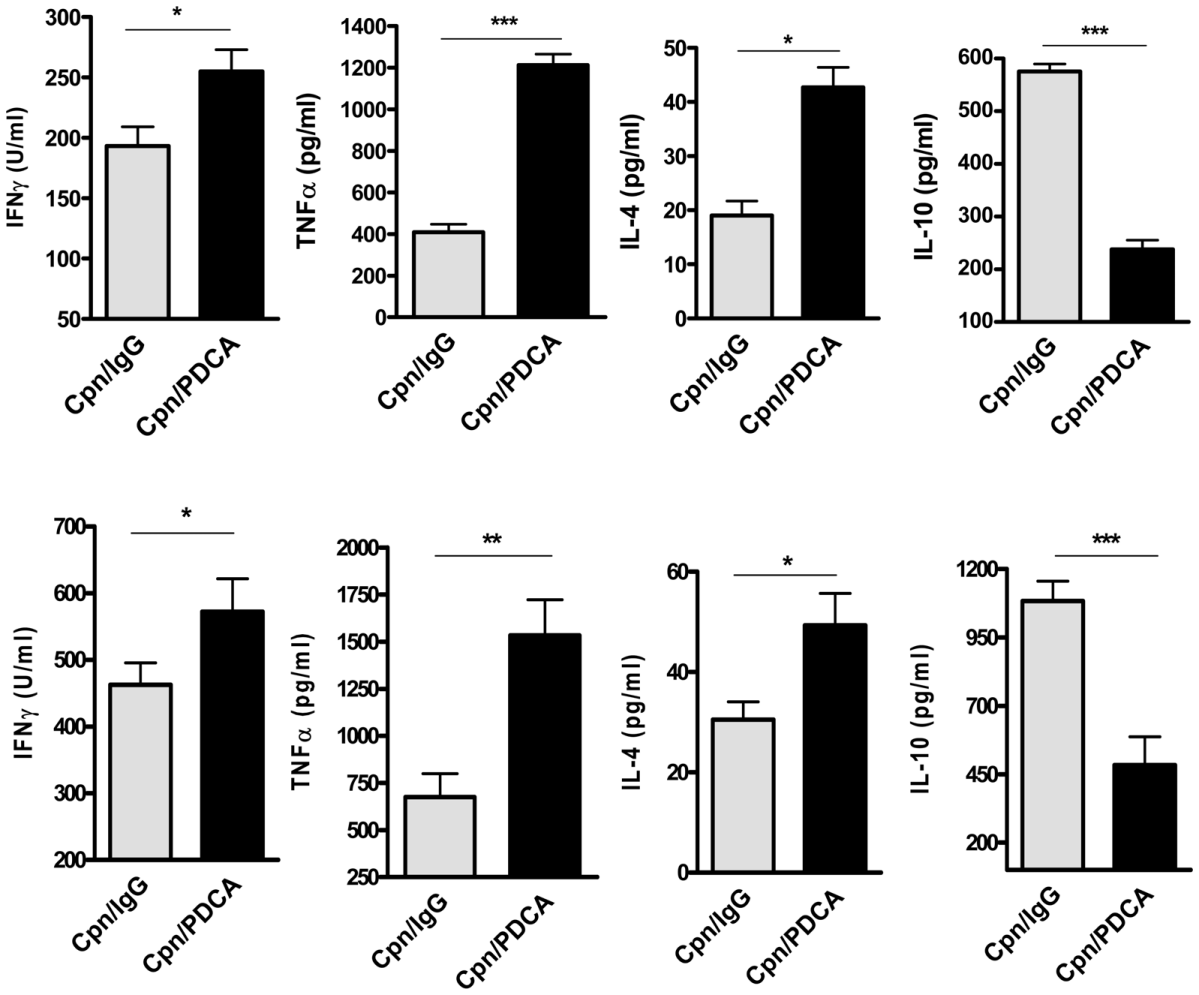
pDC deficiency *in vivo* leads to altered cytokine responses. ***A***, pDC depleted and the sham-treated mice (four mice/group), after *Cpn* infection were sacrificed 9 days postinfection and the lungs and draining (mediastinum) lymph nodes were collected. The draining lymph node (dLN) cells were cultured in the presence of SK-EB as described in *Materials and Methods*. Cytokine levels (IFN-γ, TNFα, IL-4 and IL-10) in 72-h dLN cell culture supernatants *(* ***A*** *)* and lung homogenates *(* ***B*** *)* were measured by ELISA. Data are presented as the mean ± SD of each group. Results of one of the three experiments with similar results are shown. *, *p*<0.05, **, *p*<0.01, and ***, *p*<0.001; Student’s t test.

Cytokines are key mediators of immune response and they play a central role in determining the disease outcome to either protection or pathology in chlamydial infections. To explore the basis for the increased severity of infection in pDC depleted mice, we examined the overall cytokine response in these mice by testing the *Cpn*-driven cytokine production pattern by the lung draining lymph node cells (dLN) after *Cpn* infection. Quite strikingly, dLN cells from pDC depleted mice produced significantly higher levels of IFNγ, TNFα and IL-4 compared to that from the sham-treated mice (Figure 3A). In contrast, the dLN cells from these mice produced significantly less IL-10 compared to that from the controls. Cytokine analyses in the lung homogenates also showed that pDC depleted mice had higher amounts of IFNγ, TNFα and IL-4, but lower IL-10 content in their infected lungs compared to that in the control mice (Figure 3B). The cytokines analysed were not detected in lung homogenates of uninfected mice (not shown). Overall, these results showing an altered cytokine pattern in pDC deficient condition suggested an important role for pDC in regulating the cytokine responses following *Cpn* infection.

### Pathogenic T Cell Responses Characterized by ‘Inflammatory Type’ Effector CD8 T and CD4 T Cells in the Lungs of pDC Depleted Mice Following *Cpn* Infection

**Figure 4:**
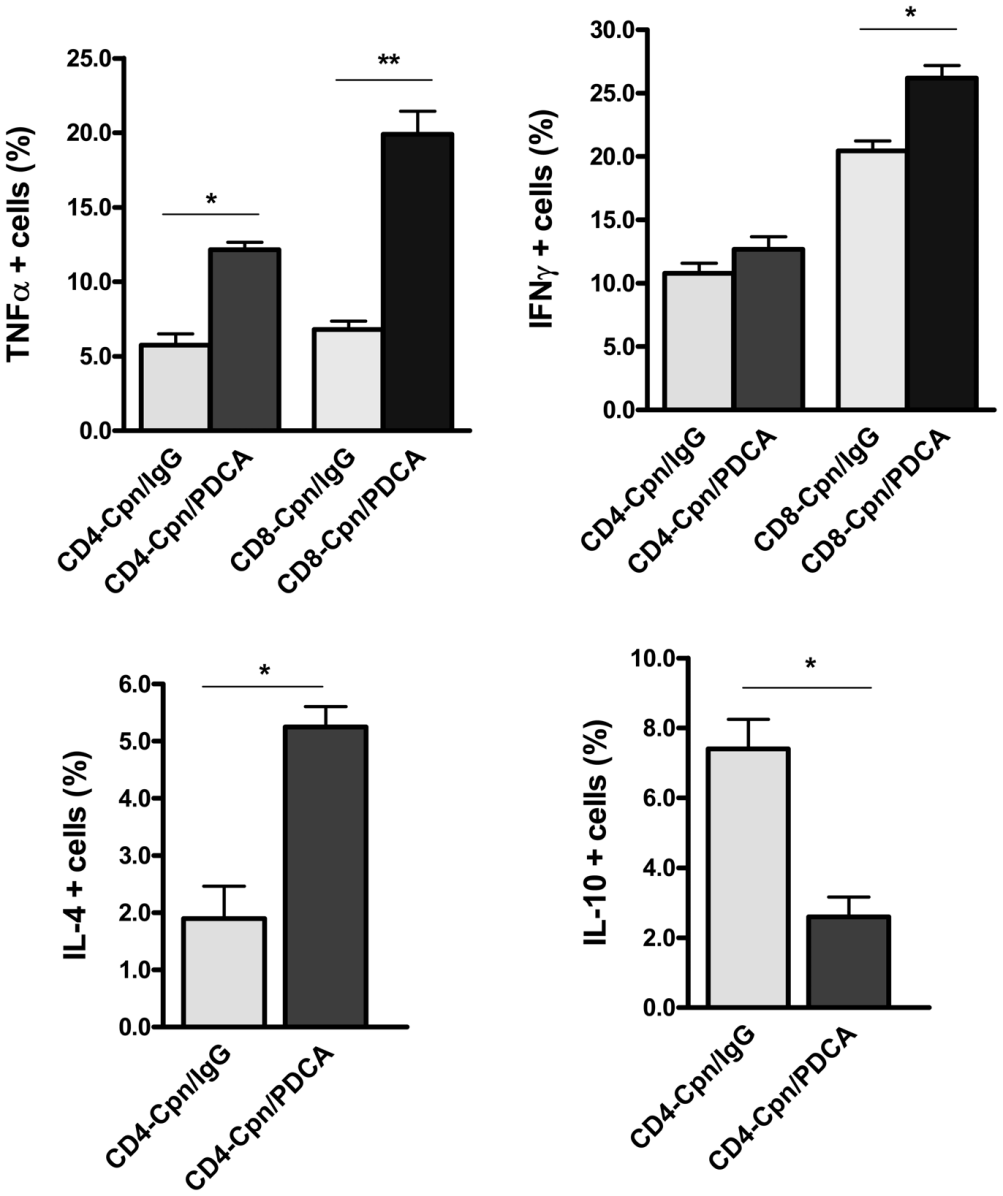
T cell specific cytokine pattern in the lungs of pDC depleted mice following *Cpn* infection. Following *Cpn* infection (day 9), the cytokine pattern of CD8 and CD4 T cells in the lungs of pDC depleted and control mice was analysed by intracellular cytokine staining. Graphs show the summary data for TNFα *(* ***A*** *),* and IFNγ *(* ***B*** *)* production by CD8 and CD4 T cells and IL-4 *(* ***C*** *)* and IL-10 *(* ***D*** *)* production by CD4 T cells. Data are expressed as mean ± SD. Three independent experiments with three mice in each group were performed and one representative experiment is shown. *, p<0.05, **, p<0.01.

Specific T cell effector responses at the local site of infection can result in either protective or pathological consequences. Therefore, we further evaluated whether pDCs influence the CD8 and CD4 T cell cytokine responses in the lungs following *Cpn* infection. The results showed that CD8 T cells of pDC depleted mice, compared to the sham-treatment group produced significantly higher TNFα and IFNγ as analysed by intracellular cytokine staining (Figure 4A, 4B). In addition, CD4 T cells from the pDC depleted mice also showed higher TNFα and IL-4 production than the sham-treated mice (Figures 4A, 4C), although the IFNγ production by CD4 T cells was not significantly different between the two groups of mice (Figure 4A). In contrast, compared with the sham-treated mice, pDC depleted mice displayed dramatically reduced IL-10 production by CD4 T cells (Figure 4D).

**Figure 5:**
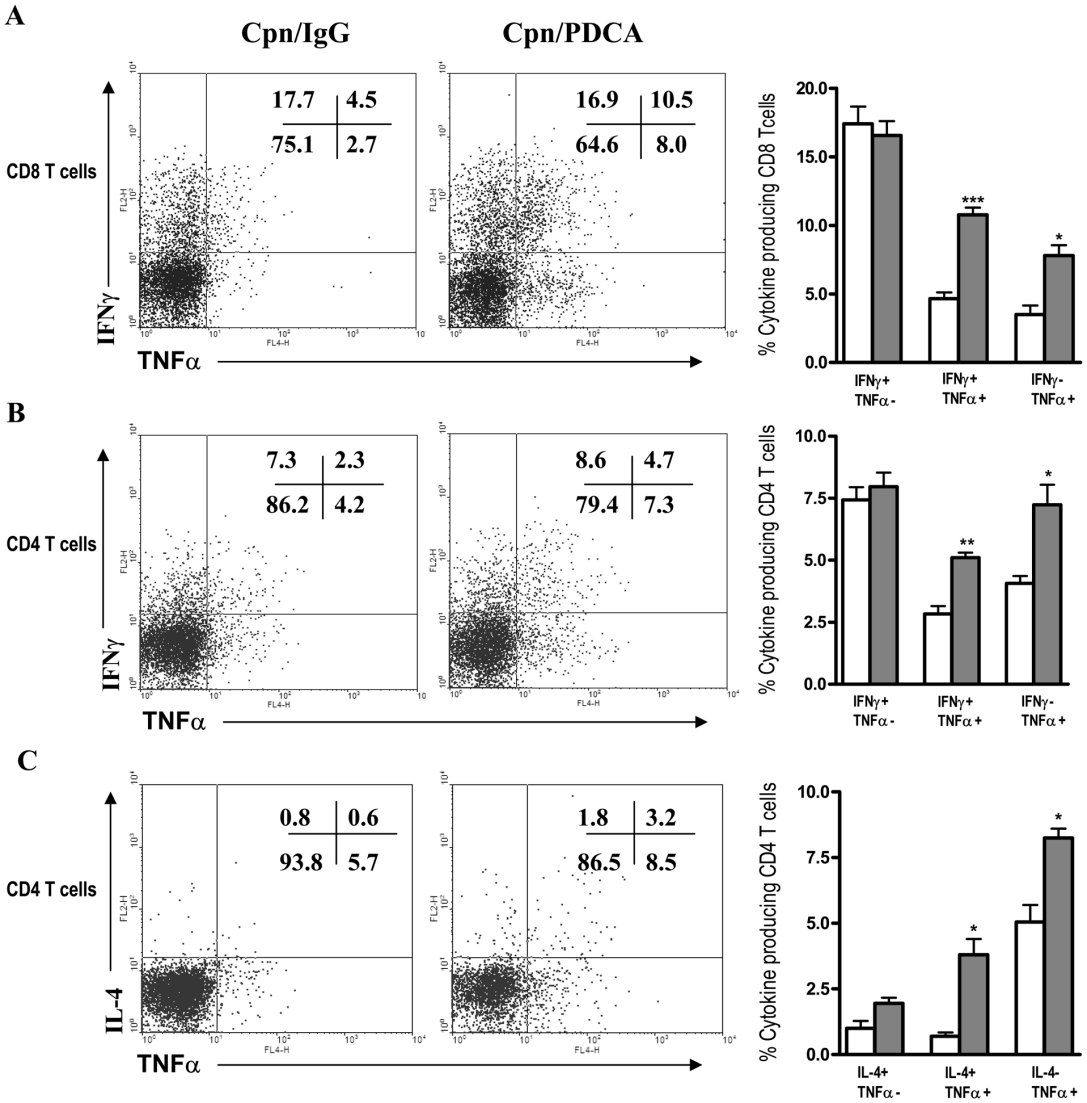
pDC deficiency leads to inflammatory type effector CD8 T and CD4 cell responses after *Cpn* infection. Lung T cells as described in the legends to Figure 4 were analyzed by multi-color intracellular cytokine staining. Shown are representative flowcytometry dot plot images and summary graphs depicting the nature of cytokine responses by T cells. Analysis was performed on gated CD3+ CD8+ cells and CD3+CD4+ cells. Note increased inflammatory type-1 (IFNγ+TNFα+) CD8 *(* ***A*** *)* and CD4 T cells *(* ***B*** *)* and inflammatory Th2 type (IL-4+ TNFα+) CD4 T cells *(* ***C*** *)* in the lungs of pDC depleted mice compared to the control group mice. At least three independent experiments were performed and the data from one representative experiment is depicted. Data expressed as mean ± SD. *, p<0.05, and ***, p<0.001.

TNF-α has been implicated as a key cytokine in many inflammatory disease conditions including respiratory diseases [21], [22]. The increased TNFα production by both CD8 and CD4 T cells in pDC depleted mice suggested an inflammatory type response by these cells. To critically examine the nature of IFNγ and IL-4 producing T cells in more detail, we further performed a double cytokine analysis with each T cell subset. Specifically, CD8 and CD4 T cells were analysed for IFNγ production along with co-staining of TNFα. We found that, in pDC depleted mice, a significant proportion of the IFNγ producing CD8 T cells also produced TNFα (more than 2 fold increased compared to that in the control mice), thus showing an inflammatory Tc1 (IFNγ+TNFα+) phenotype (Figure 5A). Similarly, CD4 T cells also showed higher proportion of IFNγ+TNFα+ (‘inflammatory Th1’) cells in the former than the latter (Figure 5B). The fraction of cells which produced IFNγ alone was strikingly similar between pDC depleted and sham-treated mice in case of both CD8 and CD4 T cells, thus showing a major difference in the amount of the inflammatory Tc1/Th1 cells between these two groups of mice. Since pDC depleted mice also showed increased IL-4 production by the CD4 T cells, we also examined for cells producing both IL-4 and TNFα (‘inflammatory Th2’) cells. As shown in Figure 5C, these mice showed significantly higher percentage of inflammatory Th2 cells (IL-4+TNFα+) compared to the controls. Together, these data demonstrated an inflammatory type-1 (CD8 and CD4) and inflammatory Th2 cell response in pDC depleted mice, suggesting that pDCs critically mediate the control of pathogenic type T cell responses following *Cpn* infection.

### Mice Depleted of pDCs Show Significant Reduction in CD4 Regulatory T Cells in the Lungs and Bronchial Draining Lymph Nodes after *Cpn* Infection

**Figure 6:**
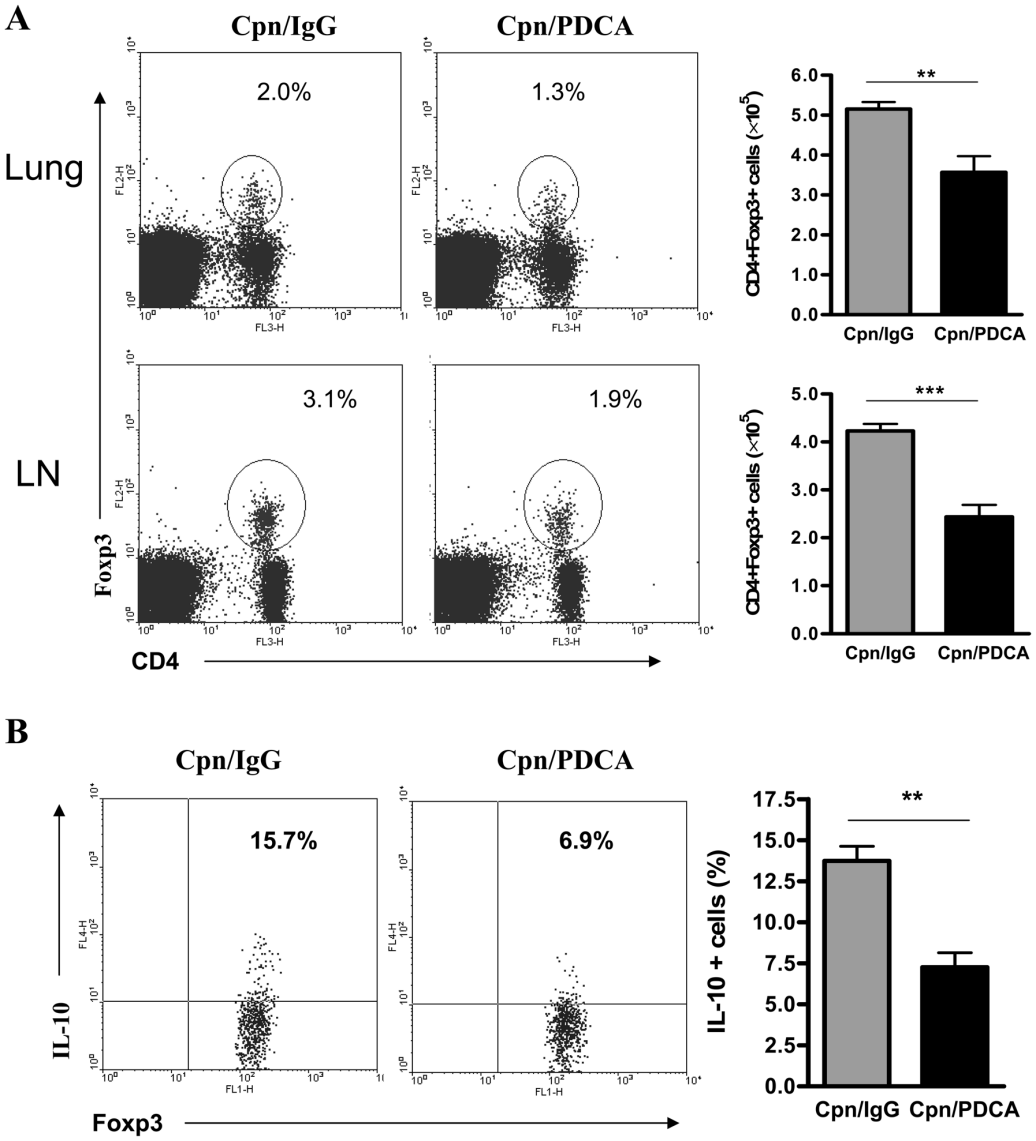
Reduced Treg numbers and lower IL-10 production by Tregs in pDC depleted mice. The frequencies of Tregs in the lungs and dLNs in pDC depleted and the sham-treated mice were analyzed following *Cpn* infection (day 9 p.i.). ***A,*** representative flowcytometry images show the percentages of CD4+Foxp3+ Tregs in the lungs and dLN**.** The absolute numbers of Tregs were depicted in the respective graphs. ***B*** *,* Intracellular staining for IL-10; The dLN cells were cultured with SK-EB for 3 days and restimulated with PMA and ionomycin. The cells were stained first for surface markers, fixed, permeabilized and stained intracellularly for IL-10 as described in *Materials and Methods.* Shown are representative flowcytometry plots and the summary graphs for IL-10 production by Tregs (gated on CD4+Foxp3+CD8− cells). Data expressed as mean ± SD. Results are representative of three independent experiments with three mice in each group. **, p<0.01, ***, p<0.001.

As shown above, the detailed analysis of cytokine pattern showed increased inflammatory Tc1/Th1 and Th2 type cytokine responses, but reduced anti-inflammatory cytokine (IL-10) production in the pDC depleted mice, that was associated with increased lung inflammation and pathology. CD4 regulatory T cells (Tregs) have been implicated in the control of inflammatory immune responses in many disease settings. Therefore, we further analysed for CD4+Foxp3+ Tregs in the lungs and draining lymph nodes of pDC depleted and sham-treated mice after *Cpn* infection. We found that mice depleted of pDCs showed very dramatic reduction in Tregs. As illustrated in Figure 6A, compared to the sham-treated mice, pDC depleted mice showed significant reduction in the frequency and numbers of Tregs in the lungs as well as dLNs.

Since it is known that IL-10 is an important effector mechanism for the regulatory activity of Tregs in the control of inflammatory response, we next analysed the difference in IL-10 production by Tregs (Foxp3+ CD4+ T cells) in these two groups of mice. Intracellular cytokine analysis showed that Tregs in the pDC depleted mice produced significantly lower IL-10 compared to that in the sham-treated mice (Figure 6B). Collectively, these data suggested that pDCs strongly influence the development and functional activity of Tregs during *Cpn* infection.

### pDCs from *Cpn* Infected Mice are Potent in Inducing Treg IL-10 Production

**Figure 7:**
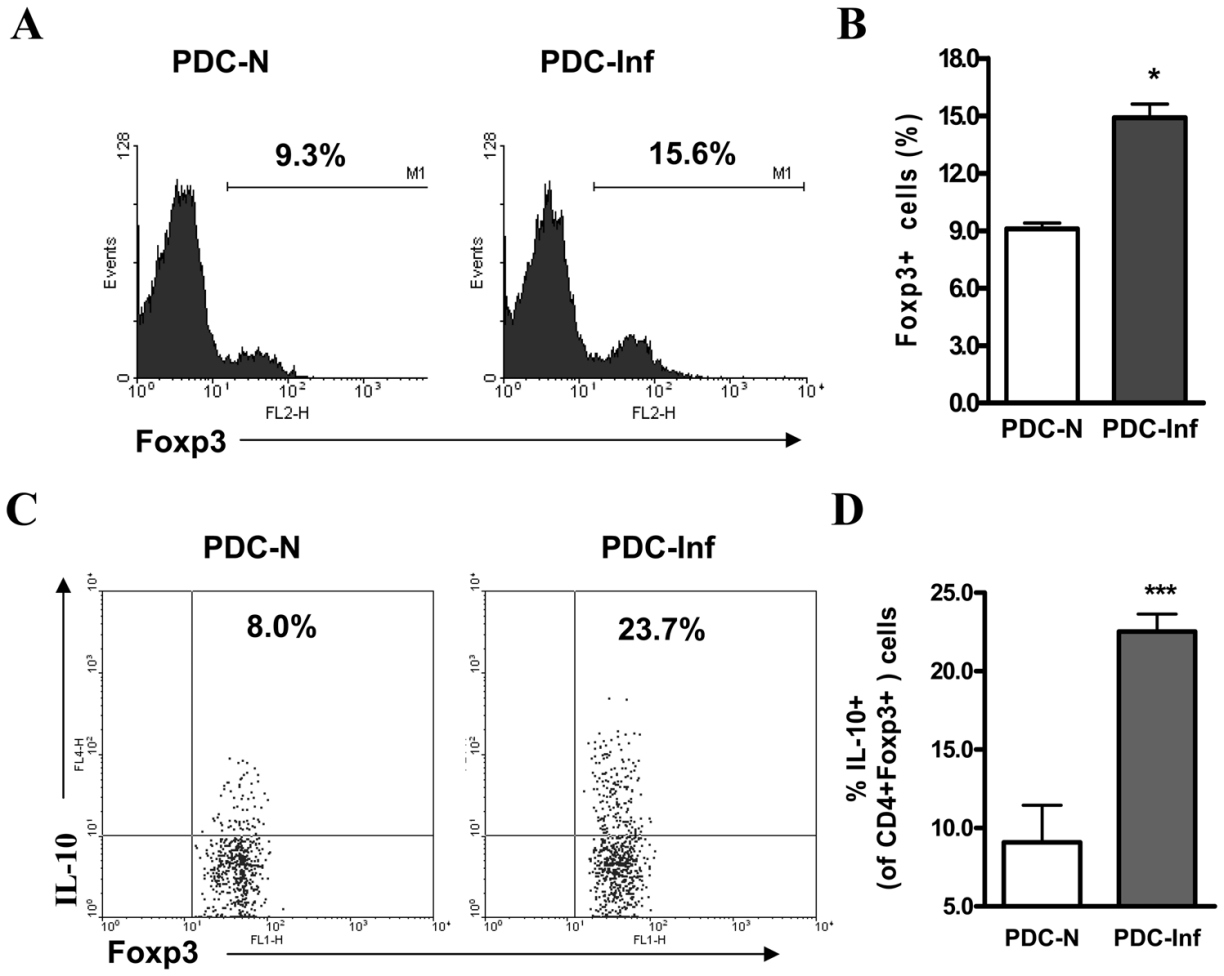
*Cpn* infection activated pDCs induce Treg IL-10 production *in vitro*. pDC isolated from the lungs of *Cpn* infected (day 9 p.i.) and uninfected mice were co-cultured with CD4 T cells purified from *Cpn* immunized mice in the presence of SK-EB as described in the *Materials & Methods.* Following three days of co-culture, the cells were washed and analysed by flow cytometry. ***A&B***, The cells were stained for intracellular Foxp3 expression. The analysis was performed on gated CD4+ cells. Representative histograms *(* ***A*** *)* are shown and the summary graph *(* ***B*** *)* provided. ***C&D,*** IL-10 production by Tregs; analysis was performed on CD4+Foxp3+ gated cells. Shown are representative dot plots *(* ***C*** *)* and a graphical summary *(* ***D*** *)* of the percentages of IL-10 producing Tregs. Data are shown as mean ± SD. Results of one representative (of three) experiment with five mice in each group are shown. *, p<0.05, **, p<0.01.

Further, we performed *in vitro* pDC-CD4 T cell co-culture experiments to directly examine the effect of pDCs to activate *Chlamydia*-specific T cell response and to confirm their ability in inducing the functional development of Tregs following *Cpn* infection. The advantage of the *in vitro* co-culture system was that only pDCs and T cells were involved, unlike the potential multiple cellular interaction which might happen *in vivo,* thus helpful for directly testing the pDC function. pDCs purified from the lungs of *Cpn* infected (day 9 p.i.) and uninfected mice were co-cultured with CD4 T cells isolated from *Cpn*-immunized mice in the presence of SK-EB stimulation and analyzed for change in Foxp3 expression and IL-10 production by Tregs. We found that pDCs from *Cpn* infected mice (PDC-Inf) compared to those from naive mice (PDC-N) induced significantly higher proportion of Foxp3+ CD4 T cells (Figure 7A&B). More importantly, we found that PDC-Inf compared to PDC-N induced significantly higher IL-10 production by Foxp3+Tregs as analysed by intracellular cytokine staining (Figure 7C&D). These data demonstrate that *Cpn* infection activated pDCs are functionally programmed to generate specific CD4 Tregs with increased IL-10 production, thus confirming the data observed *in vivo*.

## Discussion

The present study demonstrates the critical regulatory role of pDCs in host defense against *Cpn*, an important human pathogen causing acute and chronic inflammatory lung diseases. pDCs act as a regulatory mediator to check uncontrolled immune response and tissue pathology during *Cpn* infection. This is supported by the findings that *in vivo* depletion of pDCs resulted in higher inflammatory cytokine response, but decreased elicitation of Tregs and IL-10 production that was associated with extensive lung pathological changes and unresolved disease following *Cpn* infection. Importantly, the pDC deficient mice showed higher infection load compared to the pDC intact mice. These results depict a significant protective role of pDCs in an obligate intracellular bacterial infection and, in general, highlight the importance of immune regulatory mechanisms in host defense against microbial infections. These findings are very relevant for human diseases linked to *Cpn* infection. In conditions like COPD, it is the combination of impaired regulatory mechanism in the host and an exceedingly high effector T cell cytokine response that contribute to the development of chronic airway inflammation. This is consistent with the evidence that *Cpn* infection is associated with an unbalanced proinflammatory cytokine response in patients with lung inflammatory disease compared to healthy control subjects [23].

The current study on pDCs sheds light on the regulatory aspects of T cell immunity that remained unclear for chlamydial infections. Numerous studies have shown that type 1 T cell immunity is important for host protection against chlamydial infections and that Th2 responses are not protective [6]–[8], [24]–[26]. However, from the current set of data, it appears evident that Th1 or Th2 skewed responses alone do not determine the infection outcome following a series of complex and intricate immunological events occurring during infection. An important implication of the present study is that the effector T cell responses need to be regulated and optimal to fight against infection without damage to the host tissue. The findings presented here urge for a paradigm shift from the T cell type 1/type 2 balance towards the understanding of immune regulation and homeostasis for host protection against *Cpn* infection. Overall, our results emphasize the important role of regulation of host immune response for protective immunity against infections due to *Chlamydia*, given that most of the pathologies associated with these infections are immune mediated.

Similar to our findings on the effect of pDCs after *Cpn* infection, a recent study by Crother *et al* [17] previously showed that pDC depletion resulted in an impaired ability to control Cpn infection in the lungs. While these authors observed that depletion of pDCs resulted in reduced lung cellular inflammatory during acute *Cpn* infection, the mice had developed prolonged inflammation in their lungs. It was notable these authors observed reduced inflammatory cytokine production in the lungs of pDC depleted mice at the innate stage of infection, notably IFNγ, the cytokine which is critical for control of infection Cpn infection. However, they found the levels of IFNγ between pDC depleted and control mice at the later stage of infection was not different. In contrast, we found that pDC deficiency resulted in higher IFNγ production in the lungs in the adaptive immune phase following infection, although we did not analyse at early stage of infection. More over, we found a dramatically higher TNFα production along with higher IFNγ in pDC depleted mice showing a high level of inflammatory cytokine response, suggesting a mechanism contributing to pathological consequence with the deficiency of pDCs. Importantly, the bacterial loads were much higher in the lungs pDC depleted mice than the control mice. It is more likely that the overall higher production of inflammatory cytokines, IFNγ and TNFα, but lower IL-10 in pDC depleted mice (Figure 3) could be a result of uncontrolled chlamydial *in vivo* growth and a continuous over stimulation of host immune system upon the lack of regulation mediated by pDCs.

The functional role of pDC in host adaptive immunity to *Cpn* and other bacterial infections is largely unexplored. In particular, it is critical to understand how pDC modulate the T cell responses which is a key determinant in influencing the infection outcome. T cell effector responses can either lead to protective or detrimental outcomes. The present study using a bacterial respiratory infection model provides unique set of data which elucidates the nature of pathogenic type T cell responses that is elicited upon loss of pDC mediated regulation. The lungs of pDC depleted mice had significant accumulation of T cells following *Cpn* infection. With respect to *Cpn* infection, CD8 T cells have been shown to play a prominent role in protective responses [6], [8], [27]. On the other side, the damaging role of CD8 T cells is well established in lung diseases. Although our current findings show an association between increased pathogenic type CD8 T cell response and increased lung disease in pDC depleted mice, it would be important to further confirm if CD8 T cells are a critical factor for the pathological response that was observed. One approach is to deplete CD8 T cells in the pDC depleted mice at selected time points and examine whether this may mitigate the undesired immunopathology. Presumably, this strategy may still preserve the CD4 Tregs in pDC depleted mice. Recent evidence indicates that the production of proinflammatory cytokines by lung CD8 T cells contributes to pathogenesis of lung inflammatory diseases [28]. Given the tight association of *Cpn* infections with acute and chronic respiratory diseases, it would be important to dissect the underlying mechanisms that trigger pathogenic CD8 T cell responses. Specifically, our findings illustrate that the nature of CD8 T cell cytokine production was different between the pDC depleted and undepleted mice. Although pDC deficient mice showed an overall higher proportion of IFNγ producing CD8 T cells compared to that in pDC sufficient mice, subsequent characterization clearly defined the difference in the nature of IFNγ response between these groups of mice. In particular, the former, compared to the latter had increased proportion of IFNγ+TNFα+ (double positive) CD8 T cells (Figure 5A). Notably, IFNγ+TNFα− (single positive) cells were similar between pDC depleted and control mice (Figure 5A). It is likely that these single positive cells are the desired type of cells contributing to the ‘right’ type-1 T cell response needed for protection against *Cpn* infection, while the double positive cells are pathogenic. The excessive production of TNFα in pDC depleted infected mice associated with lung inflammation is in line with several studies that have shown the adverse effect of TNFα in different pathogenic conditions including infections [21], [22], [29]. It could be speculated that pDCs balance the IFNγ: TNFα ratio in T cell response during *Cpn* infection. Notably, we also found that a significant proportion of IL-4 producing CD4 T cells in the pDC depleted, infected mice were IL-4+ TNFα+ cells. While it is known that Th2 responses are non-protective in chlamydial infections, the nature of pathogenic Th2 cells has not been characterized. Our present findings provide a lead for better understanding on the nature of Th2 cell responses that can cause pathological events during *Cpn* infection. Further studies are needed to address the precise role of inflammatory Th2 cells in chlamydial pathogenesis.

Another key finding in this study is the demonstration of the functional role of pDCs in eliciting Treg activation/IL-10 production that is correlated with protection against immune mediated pathology following *Cpn* infection. First, pDC depletion resulted in dramatically lower numbers of Tregs in the lung, the local infection site and also in the draining lymph nodes (Figure 6A). These results were further supported by the findings from experiments using the *in vitro* pDC-CD4 T cell co-culture model. pDCs isolated from *Cpn* infected mice were very potent in inducing specific Tregs secreting IL-10. The ability of pDCs to induce Tregs has been previously shown in numerous disease settings [10], [30], [31]. Importantly, our present findings using *Cpn* lung infection model depict the critical role of regulatory network by pDCs and Tregs and provide an insight into the linkage of immune regulation and development of protective adaptive immune response in host defense against real infections. Tregs restrain variety of immune responses, partly through production of IL-10 [32]–[34]. The induction of IL-10 producing Tregs is associated with a beneficial outcome to the host in this infection model. This is supported by the data that the reduction of Tregs and IL-10 production in pDC depleted mice is correlated with histologic findings showing massive cellular infiltration and extensive tissue damage in their lungs after infection. IL-10 has been shown to inhibit immunopathological consequences in certain infection models [35], [36], while its effect is detrimental in some other infections [37], [38]. It is worth recalling that IL-10 has been linked to susceptibility in earlier chlamydial studies, specfically *C. trachomatis* infection [39], [40]. Typically, while IL-10 can limit tissue immunopathologies, it can also slow down pathogen clearance. In agreement with this notion, a recent study by Penttila *et al* showed accelerated clearance of *Cpn* infection, but enhanced pulmonary pathology in IL-10 KO mice [41]. Our finding showing reduced IL-10 production in pDC depleted mice associated with increased lung pathology after *Cpn* infection advocate a beneficial role for IL-10, at least partially in this model. Nevertheless, we believe that the kinetics of IL-10 production is critical in determining the protective or non-protective outcome. It is possible that during infection, the cellular source of IL-10 may dictate its cellular target and outcomes. Further studies are needed to address the kinetics of Tregs/IL-10 in the context of infections. It is notable that pDC-depleted mice had pronounced T cell responses, but markedly increased levels of infection in the lung. A limitation of the present study is that it has examined most of the readouts at a single time point, i.e at day 9 p.i., at the adaptive immune phase. Analysis of the earlier time points could have derived information if the cellular immune responses in the lung might be delayed in pDC-depleted mice during the innate phase. In addition, further studies are needed to precisely characterize the dynamics of the T cell/Treg responses which can provide better understanding of regulatory mechanisms during the process of infection. It has been shown that TLRs, especially TLR2 and 4 regulate IFNγ producing T Cells during *Cpn* pulmonary Infection. Moreover, increase in IFNγ release by T cells in the absence of both TLRs was shown to be associated with a reduced frequency of Tregs [42]. Considering the present study results showing increased IFNγ producing T cells, but reduced Tregs in pDC depleted mice, it would be important to address whether TLR2/4 related mechanisms are involved in the deficiency of pDCs in this model. pDC regulatory mechanisms may differ with respect to the type of infections. In this model of an intracellular bacterial infection, it is unlikely that pDCs contribute to host defense through an IFNα mediated mechanism (well known in most viral infections). The IFNα production by pDCs was similar between *Cpn* infected mice and uninfected mice (Figure 1C). In addition, there was no difference between the IFNα mRNA levels by PCR in lungs of *Cpn* infected, pDC depleted mice and the sham-treated mice (data not shown). This is in line with the observations in certain infections that, the contribution of pDC derived IFNα is limited or dispensable for pathogen clearance and protection [14], [43], [44].

In addition to its significance in elucidating the mechanism regarding immune regulation against *Cpn* infection, this study has significant implications in our understanding of the host defense against infectious diseases with respect to pDCs. The present findings demonstrate that pDCs, which are classically thought of as antiviral, play an important role in host control of an intracellular bacterial infection. Perhaps, these findings may not be unique to *Cpn* infection, but may also extend to other intracellular bacterial infections such as tuberculosis and Lyme’s disease. It is also possible that pDCs may even play a role in host responses to intracellular parasite infections, for e.g., malaria and leishmania. The present findings also raise questions regarding the varying functional roles of pDCs in the regulation of cellular responses in different infections. For example, our results obtained using this intracellular bacterial lung infection model point to a regulation of heightened CD8 T cell responses by pDCs during *Cpn* infection. In contrast, in certain viral infections such as MCMV, pDCs support for an enhanced accumulation and effector function of CD8 T cells [45]. Thus, pDC mediated functions can manifest in different ways in host immune protection with respect to the type of pathogens and infections. In a broad sense, our study showing a severe disease outcome in pDC deficient mice exemplifies how defects in cellular control of T cell (CD8 and CD4) responses can exacerbate lung inflammatory conditions after infections, and show the critical role and mechanism by pDCs in the regulation of these responses. Clearly, more studies on pDCs in different type of infections are a priority to provide broadened knowledge on the dynamic role of these cells and mechanisms contributing to immunity against various pathogens.

In conclusion, our findings provide direct *in vivo* evidence for a critical protective role of pDCs in homeostatic regulation of immunity by controlling inflammatory type T cell responses following respiratory infection due to *Cpn*, a medically important bacterial pathogen. These data represent an example showing that the delicate balance in regulation of immune response can be lost upon a pDC deficient condition leading to the progression of infection with severe inflammatory disease. Our overall findings highlight the importance of a ‘balanced’ immune response for host protective immunity and preventing detrimental immunopathology in microbial infections. Further studies on pDC mediated mechanisms will provide deeper understanding on the linkage between immunoregulation and development of optimal host immunity against infectious diseases.
